# Re-defining reproductive coercion using a socio-ecological lens: a scoping review

**DOI:** 10.1186/s12889-023-16281-8

**Published:** 2023-07-17

**Authors:** Melissa Graham, Greer Lamaro Haintz, Megan Bugden, Caroline de Moel-Mandel, Arielle Donnelly, Hayley McKenzie

**Affiliations:** 1 Department of Public Health, School of Psychology and Public Health, La Trobe University, 3086 Bundoora, VIC Australia; 2 School of Health and Social Development, Deakin University, Locked Bag 20, Geelong, 000, 3220 Australia; 3 School of Health and Social Development, Deakin University, 221 Burwood Highway, Burwood, 3125 VIC Australia

**Keywords:** Reproductive coercion, Scoping review, Reproductive autonomy, Definitions

## Abstract

**Background:**

Reproductive coercion is a significant public health issue in Australia which has mainly been conceptualised as a form of violence at the interpersonal level. This limited scope ignores the role of the gendered drivers of violence and fails to encompass a socio-ecological lens which is necessary to consider the multiple interacting layers that create the context in which reproductive coercion occurs. The aim of the scoping review was to explore how the reproductive coercion is defined by international research. Specifically, how is reproductive coercion defined at the social-cultural-systems-structural levels, and are the definitions of reproductive coercion inclusive of the conditions and contexts in which reproductive coercion occurs?

**Methods:**

A scoping review was undertaken to explore existing definitions of reproductive coercion. Searches were conducted on Embase, Cochrane Library, Informit Health Collection, and the EBSCOHost platform. Google was also searched for relevant grey literature. Articles were included if they were: theoretical research, reviews, empirical primary research, grey literature or books; published between January 2018 and May 2022; written in English; and focused on females aged 18–50 years. Data from eligible articles were deductively extracted and inductively thematically analysed to identify themes describing how reproductive coercion is defined.

**Results:**

A total of 24 articles were included in the scoping review. Most research defined reproductive coercion at the interpersonal level with only eight articles partially considering and four articles fully considering the socio-cultural-systems-structural level. Thematic analysis identified four main themes in reproductive coercion definitions: Individual external exertion of control over a woman’s reproductive autonomy; Systems and structures; Social and cultural determinants; and Freedom from external forces to achieve reproductive autonomy.

**Conclusions:**

We argue for and propose a more inclusive definition of reproductive coercion that considers the gendered nature of reproductive coercion, and is linked to power, oppression and inequality, which is and can be perpetrated and/or facilitated at the interpersonal, community, organisational, institutional, systems, and societal levels as well as by the state.

**Supplementary Information:**

The online version contains supplementary material available at 10.1186/s12889-023-16281-8.

## Introduction

Reproductive coercion is a significant public health issue globally, driven by gender inequality and imbalances in power, with negative consequences for a wide range of sexual, reproductive, and mental health issues [1–2]. It is important to acknowledge the historical context of women’s reproduction whereby extended family members, health professionals, and the state have all limited women’s reproductive rights and autonomy [3]. In several countries, including Australia, colonisation has also had a role in compounding forms of reproductive coercion occurring at a structural level, facilitated by the state [4, 5]. For example in Australia, Aboriginal and Torres Strait Islander women and girls were subjected to assimilationist legislation and policies by the State which resulted in the Stolen Generation, wherein infants and children were forcibly removed from their families and their communities [3, 4, 6]. Forced marriage and forced sterilisation were also common practices [3]. Reproductive coercion has been experienced by multiple diverse groups including, but not limited to, single/unwed mothers, women and girls with disabilities [7], intersex people, and women from newly arrived, refugee and migrant backgrounds [3, 6]. Further, both historically and currently, women in rural and remote areas or women with low socioeconomic status are disproportionately impacted by state policies which create limited access to affordable contraception and abortion, constraining women’s autonomy to enact their fertility desires [3]. This failure to focus on how structural factors within society institute reproductive coercion renders diverse groups of Australian women’s experiences invisible.

Given the historically hidden nature of reproductive coercion, limited Australian research exists to demonstrate its full extent. The data that does exist relates to women’s experiences of gender-based violence, which commonly includes reproductive coercion. Research exploring the prevalence of violence against women in Australia demonstrates that one in five women over the age of 15 will experience violence in their lifetime [8]. Further, a recent study found 15% of women attending two family planning clinics had experienced reproductive control and abuse, which included pregnancy prevention and abortion [9]. Public health practice widely accepts that gender inequality is the underlying cause of all forms of violence against women [10–14]. Research exploring the determinants of violence against women in Australia is ongoing, but at present suggests gender norms and structural gender inequality interact in complex ways, across social ecology, to influence the prevalence of violence against women through long causal pathways [15, 16].

The categorisation of reproductive coercion as a form of violence [17, 18] has meant it has predominantly been addressed within a prevention of violence against women framework, focusing on the interpersonal level where tertiary prevention (such as services responding to violence and supporting survivors) occurs. Prevention at this level is essential and important advocacy work has been done by many feminist organisations and academics over the past decades to ensure reproductive coercion is appropriately recognised as a form of violence occurring at this level. While this has enabled effective identification and response to reproductive coercion at the interpersonal level, a failure to recognise and identify reproductive coercion occurring at other levels can lead to women’s experiences of violence being invisible and impacts primary prevention efforts. One such impact of this is the neglect of policy development and reform addressing reproductive coercion in its entirety. Public health prevention needs to occur across multiple prevention levels for real and meaningful social change to occur. Therefore, a sole focus on the interpersonal level fails to consider the importance of upstream primary prevention and, in the context of violence prevention, ignores the role of the gendered drivers of violence that exist at a societal, system, institutional, organisational, and community level.

Reproductive coercion has historically been the responsibility of multiple, overlapping sectors. These include the domestic violence, sexual assault/harassment and sexual and reproductive health sectors which are informed by their own distinct approaches to prevention, policy reform and service delivery [6]. A recent enquiry into the nature and extent of reproductive coercion within Australia [6] identified eight key themes including, but not limited to, the need to address gender inequality as an underlying driver of reproductive coercion, and the need to explore the structural drivers of reproductive coercion, in addition to how reproductive coercion intersects with forms of violence at the interpersonal level [6]. This enquiry highlights the need to move beyond conceptualising reproductive coercion at the interpersonal level, which is the premise of this scoping review.

A socio-ecological approach can be utilised to move beyond the interpersonal level. This approach recognises the broader systems that individuals are embedded within and the changing nature of the social environment [19]. Socio-ecological models assert that change needs to happen at all levels to be effective and sustainable. To capture how gender norms, practices and structures underpinning violence against women exist and are maintained within and across complex layers of our social, cultural, political, and economic environments, Our Watch [12] created a shared framework which utilises a socio-ecological model to frame the primary prevention of violence against women within Australia. The framework acknowledges there are four gendered drivers of violence resulting from gender inequality which, from an intersectional lens, intersect with other forms of oppression to compound some women’s experiences of violence. These gendered drivers are the “condoning of violence against women; men’s control of decision-making and limits to women’s independence in public and private life; rigid gender stereotyping and dominant forms of masculinity; and male peer relations that emphasise aggression, dominance and control” [12](p36). The Our Watch [12] model positions the individual and relationships at the centre, with an additional three levels filtering out: organisation and community, system and institution, and societal. These levels of the model are explored specific to the structures, norms and practices that exist within them. Like popular ecological models utilised to understand and address violence against women [20, 21], the Our Watch socio-ecological model focuses on how societal level factors (specifically of gender inequality) are maintained and reinforced to create the necessary context for violence against women to occur within [12]. For example, at the individual and relationship level, hegemonic gender norms become embedded in personal identities shaping people’s understandings of the roles, value and power assigned to people based on the rigid gender binary [16]. At the organisational and community levels, norms, practices and structures within workplaces, sporting clubs, schools and other settings can support or fail to address gender inequality maintaining gender discrimination within these settings [16, 22]. At the systems and institutional level, failure to promote women’s economic, legal and societal autonomy for example, through failing to address systemic issues of the gender pay gap or the burden of unpaid care work being predominantly shouldered by women, limits women’s earnings and financial freedom throughout their lifespan compared to men thus preventing gender equality [16, 22−24]. At the societal level, dominant social norms supporting hegemonic gender are maintained and violence against women is downplayed [16], for example, through media and it’s reporting of women’s experiences of violence and failure to hold men accountable [12, 25].

The model highlights how violence against women is the outcome of interactions between various levels (particularly at the primary prevention level), therefore it uniquely moves beyond just looking at the interpersonal level (or tertiary prevention level). The model was developed to support public health practitioners and academics to approach violence against women from a primary prevention lens, which requires underlying drivers to be addressed across all socio-ecological levels. Like other forms of violence against women, we argue reproductive coercion is a complex public health issue that needs to be viewed and addressed from a socio-ecological lens, as opposed to only focusing on the interpersonal level.

It is clear reproductive coercion within the Australian context is multi-layered, complex, gendered, driven by gender inequality and differentials in power, and rooted in historical experiences yet there has been no exploration of how definitions of reproductive coercion may be limiting the scope of work to prevent and address reproductive coercion. As such, the current scoping review explored existing definitions of reproductive coercion, drawing on international research due to the limited Australian research, and asked; how is reproductive coercion defined, how is reproductive coercion defined at the social-cultural-systems-structural levels, and are definitions of reproductive coercion inclusive of the conditions and contexts in which reproductive coercion occurs? This approach enabled an exploration of reproductive coercion in the broadest sense to enable alternative definitions and contexts to be considered in how we might better conceptualise reproductive coercion in Australia. The outcome of this review is a proposed definition of reproductive coercion that moves beyond the interpersonal and considers the role of gendered drivers at the societal, system, institutional, organisational and community levels is conceptualised, which may be applicable in other similar socio-cultural-politico contexts to Australia.

## Methods

A scoping review was undertaken following the process as described by Arksey and O’Malley [26], and advanced by Levac et al. [27], and the Joanna Briggs Institute guidance for conducting scoping reviews [28]. The Preferred Reporting Items for Systematic reviews and Meta-Analyses (PRISMA) extension for scoping reviews (PRISMA-ScR) checklist [29, 30] was used to structure the current scoping review. A scoping review was most appropriate as the current review aimed to explore the extent, range, and nature of the evidence regarding definitions of reproductive coercion and identify gaps in the evidence on definitions [30].

### Search strategy

Five authors (MG, HMK, GLH, MB and CDM) developed the search strategy, search terms and eligibility criteria, which were refined after preliminary searches were completed (AD). The information sources searched included Embase, Cochrane Library, Informit Health Collection, and the EBSCOHost platform (MEDLINE, CINAHL (nursing and allied health), PsycINFO (psychology), and SocINDEX (sociology)). Google was also searched for grey literature.

Search terms covered two key concepts: (1) reproductive coercion; and (2) definition (Table 1). Search terms were developed initially through a group brainstorming process and a review of prior scoping reviews that had similarly aimed to explore definitions of concepts, followed by preliminary searches in the identified databases. Preliminary searches involved each term being entered into each database separately to ensure all terms in the final search strategy yielded results; if a term did not produce results, it was removed. Relevant MeSH terms were also considered related to both key concepts (conducted using Cochrane). All potential search terms were then reviewed and refined by the research team.

**Table 1:** Search terms

Concept 1	Concept 2
Reproductive coercion	Definition
Coercive reproduction	Conceptualise
Reproductive pressure	Framing
Reproductive control	Theory
Reproductive sabotage	Defining
Reproductive autonomy	Theoretical
Reproductive agency	Lens
	Positioning
	Constructs
	Elements
	Attributes

The search strategy was conducted by searching for all search terms individually for concept 1 (reproductive coercion) and combined using the OR operator and repeated for concept 2 (definition). Concepts 1 and 2 were then combined using the AND operator to conduct the final search. For example, “reproductive coercion” OR “coercive reproduction” OR “reproductive pressure” OR “reproductive control” OR “reproductive sabotage” OR “reproductive autonomy” OR “reproductive agency” AND definition OR conceptualise OR framing OR theory OR defining OR theoretical OR lens OR positioning OR constructs OR elements OR attributes. Searches were set to identify key terms in the titles and abstracts.

A search was also undertaken using Google to identify any grey literature. The Google search was restricted to .gov, .org, or .edu domains and was conducted using the same search terms (Table 1). The first 200 results were included in the screening process to ensure that all relevant material was searched.

### Eligibility criteria

To be eligible for inclusion articles had to be: theoretical research articles, reviews (for example, literature, scoping, rapid, systematic), empirical primary research, grey literature (for example, peak body and governmental reports), books, commentaries or opinion pieces; published between January 2018 and May 2022; written in English; full-text available; focus on females of reproductive age (18 to 50 years); and include a definition of reproductive coercion. Dissertations and conference presentations were excluded. The search was limited to articles published between January 2018 and May 2022 to ensure contemporality of the evidence.

While reproductive coercion is not limited to cis-gendered females of reproductive age, research focusing only on adolescents was excluded. However, research which included both adolescent and adult females was included. The reproductive age span selected (18 to 50 years of age) reflects when adult females are most likely to be directly impacted by and experience reproductive coercion. Reproductive coercion may also occur among transgender and gender diverse people. While non-cis-gendered female search terms were not included, the term female was not considered exclusively as meaning cis-gendered female and as such did not exclude adult female identifying populations. To this end, females were not considered exclusively as cis-gender, and reproductive age sometimes included females younger than 18 years alongside those aged 18 years and over.

### Evidence selection

Results from the searches were uploaded into Covidence. All publications were screened for eligibility. Abstracts were reviewed against the eligibility criteria by any two of the researchers. In cases of a disagreement, a third researcher reviewed the abstract which was then discussed to reach a consensus. Publications which met the eligibility criteria based on abstract screening were then moved to full-text screening. As per abstract screening, the full-text of the publications were reviewed against the eligibility criteria by any two members of the research team, with a third reviewing the full-text when there was a conflict. The three researchers then discussed the publication to reach a consensus regarding its inclusion or exclusion. The main reason for conflict was whether key findings related to defining reproductive coercion.

### Analysis

Data extraction was undertaken for each article including author(s), year of publication, publication type, aims/objectives, study design, sampling method(s) or eligibility criteria, data collection methods, study population (sample size and characteristics), key findings related to the research questions, conclusion/recommendations, and limitations. An abbreviated data extraction chart is provided in supplementary Table 1. Each article was read and re-read to extract relevant data with only data relevant to the definitions of reproductive coercion extracted into a Microsoft Excel spreadsheet. Data extraction was completed independently (AD) and reviewed by the research team. Preliminary coding was undertaken by MG and GLH by independently coding each segment of data extracted. The codes were then combined and discussed by MG and GLH in relation to their meaning and the research questions. An iterative process was then undertaken to collapse the codes into themes and refine the themes. The themes were then reviewed and further refined by the research team.

Data were thematically analysed to identify key ideas, concepts, and factors relevant to the research questions. These were then reviewed to identify any similarities and were merged to develop themes. While data extraction provided a way to draw out data, thematic analysis provided a more nuanced and contextual understanding of the ways in which reproductive coercion is defined and we believe, more informative insights than a profile of the included studies as a data chart [26, 28]. Throughout the following results section, the use of single quotation marks captures the language used in the articles included in the scoping review.

## Results

**Fig. 1:**
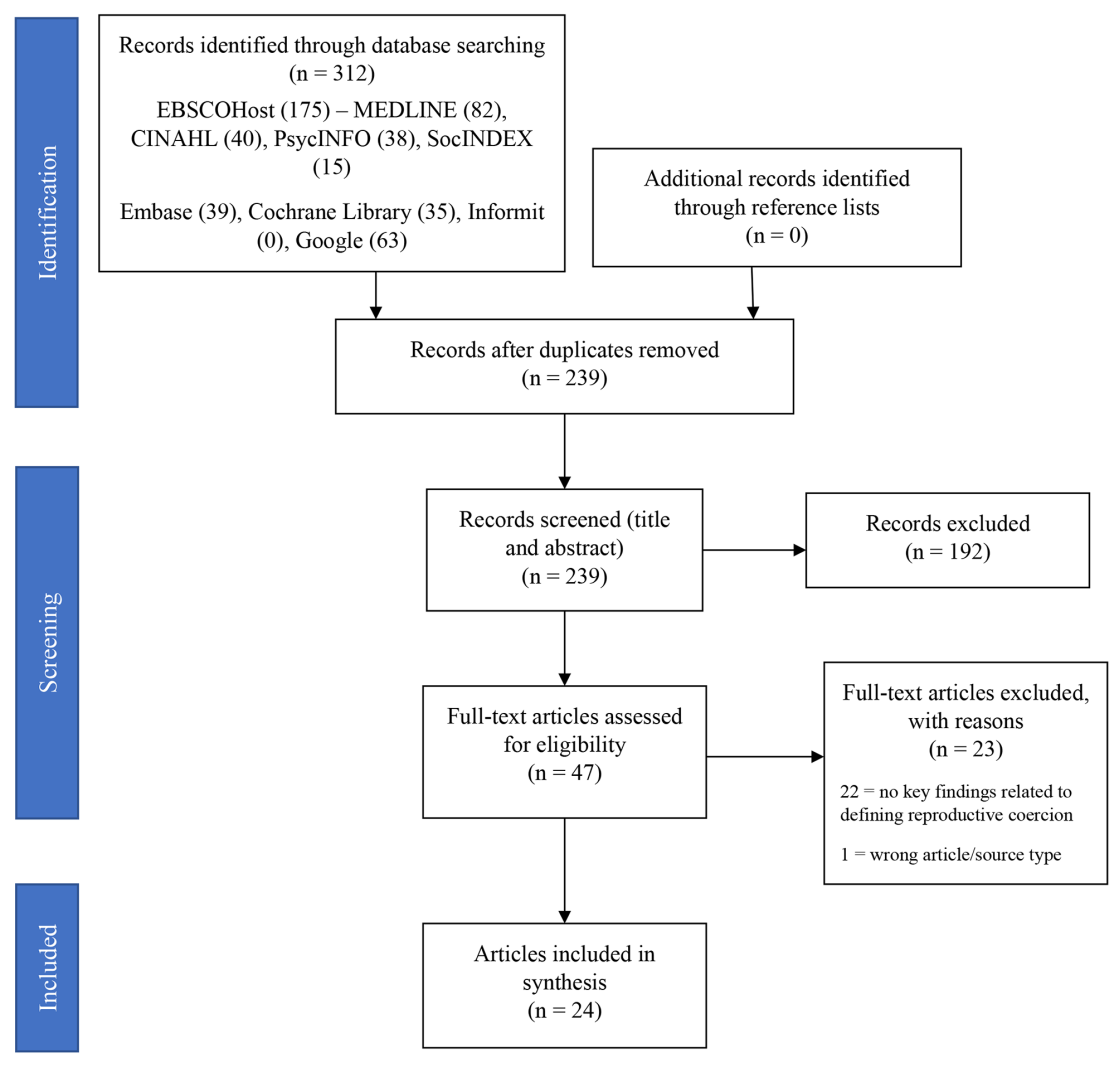
PRISMA flow diagram illustrating the evidence selection process

The search strategy produced 239 articles once duplicates were removed. Of the 239 articles, title and abstract screening against eligibility criteria excluded 192 articles. At full-text screening, 47 articles were assessed against eligibility criteria and 23 were excluded. Of the 23 excluded articles, 22 had no key findings related to defining reproductive coercion, and one article was an excluded source type (conference abstract). A total of 24 articles were included in the scoping review. Figure 1 represents the evidence selection process.

### Study characteristics

All 24 articles included study populations that focused on the experiences of females. Ten studies included females aged between 15 and 49 years [31–40], three included females who did not complete high school [31, 32, 36], six included Indigenous females [31, 33, 35, 38, 41, 42], three included female migrants [31, 32, 42], and two studies included females living with disability [38, 43]. Three studies specified the sample had to have experienced intimate partner violence [31, 42, 44]. One study included only females who had children or were pregnant [31]. Ten studies were conducted in in Australia [18, 31, 35, 38, 42, 44−48], nine in the United States [32–34, 37, 39, 40, 49−51], and one in each of the Netherlands [52], Canada [41], the United Kingdom [53], sub-Saharan Africa [36] and South Africa [43].

Twenty-one journal articles, two policy submissions [45, 46], and one discussion paper [47] were included in the review. Of the included journal articles, nine were qualitative [31–34, 36, 38, 41, 44, 48], three quantitative [35, 37, 40], four narrative reviews [43, 50, 52, 53], one systematic review [51], one qualitative evidence synthesis [42], one quantitative research proposal [39], one commentary [18], and one editorial [49]. A range of sampling methods were used in the 12 primary studies: four used purposive sampling [32, 33, 36, 48]; three used a combination of purposive and convenience sampling [31, 38, 44]; three used voluntary response [34, 37, 40]; one used convenience sampling alone [35]; and one used relational sampling [41](p4).

Sixteen studies specified their data collection methods. Eight conducted interviews including semi-structured [31, 32, 44, 48], unstructured [38, 41], and in-depth interviews [34, 36] with two also including focus groups [31, 34]; three conducted an online survey [37, 39, 40]; one used organisational data from clients [35], and one used the Delphi method [33](p12). Additionally, of the five included reviews, three searched databases and provided details on their search methods [42, 51, 53].

Three studies specified their sample eligibility criteria [42, 51, 53]. Among these there was a focus on heterosexual relationships, reproductive coercion perpetrated by men towards women, and reproductive coercion in the context of intimate partner violence.

### Reproductive coercion defined

At the most basic level reproductive coercion can be understood as anything that may impact reproductive choices and autonomy. Most research defines reproductive coercion as ‘behaviour’ that occurs at the interpersonal level (usually within an intimate relationship and/or families) ‘interfering’ with women’s reproductive ‘autonomy’ through acts such as contraceptive ‘sabotage’, ‘coercion’ to get pregnant, or ‘controlling’ the outcome of a pregnancy. Two articles [18, 47] specifically argued against defining reproductive coercion beyond the interpersonal level. Rather they posit social-cultural-systems-structural level factors are not reproductive coercion, despite acknowledging these are contributing factors which inhibit reproductive autonomy. Of the articles that did consider more than the interpersonal level, eight included definitions of reproductive coercion that were at least partially inclusive of the socio-cultural-systems-structural level [33, 34, 36, 41, 43, 46, 49, 52], for example socio-cultural norms and practices, service provision and access, policies, law, and legislation that restrict reproductive autonomy. While four articles [33, 36, 41, 52] fully considered the socio-cultural-systems-structural level, for example positioning reproductive coercion within a broader lens of reproductive justice, human and/or reproductive rights, or reproductive autonomy. Reproductive coercion was also considered in one article to be multi-directional involving ‘downward’ coercion and ‘upward’ coercion [36] and occurs across a continuum of time and/or at different phases of the reproductive lifespans.

**Fig. 2:**
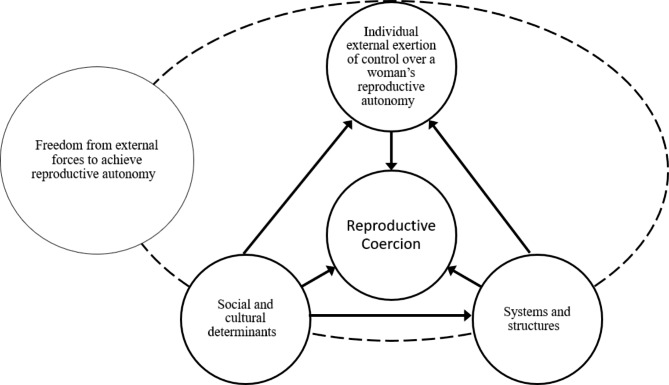
Interconnected key concepts in definitions of reproductive coercion

Thematic analysis identified four main themes which describe the ways in which reproductive coercions is defined: Individual external exertion of control over a woman’s reproductive autonomy; Systems and structures; Social and cultural determinants; Freedom from external forces to achieve reproductive autonomy. The relation between these themes is depicted in Fig. 2 and described in the following sections.

### Individual external exertion of control over a woman’s reproductive autonomy

The theme *Individual external exertion of control over a woman’s reproductive autonomy* captured the interpersonal level of reproductive coercion. Most definitions of reproductive coercion (16 articles) were positioned at the interpersonal level [18, 31, 32, 35, 37–40, 42, 44, 45, 47, 48, 50, 51, 53]. From this perspective reproductive coercion is externally exerted on a woman by others (predominantly male partners and family) driven by individual behaviours and attitudes.

Within this frame, reproductive coercion is contextualised as a form of intimate partner violence, usually perpetrated by a man against a woman, with reproductive coercion just ‘one type of coercive behaviour used by some abusers’ [32](p248). As such, reproductive coercion is considered to co-exist with sexual violence, physical, emotional, and psychological abuse, and financial control [44]. Furthermore, these forms of abuse and violence also contribute to constituting reproductive coercion independently and interactively. For example, perpetrators use these forms of abuse and violence singularly or in combination such as by exerting ‘fear’ [18, 39], ‘control tactics’ [36, 38], and ‘coercive control’ [31, 32, 44] to control a woman’s reproductive autonomy. Within these definitions reproductive coercion may include but is not limited to ‘verbal threats’ [31, 39], ‘pressure’ [44, 45, 51], threats of or actual violence by an intimate partner or family member to achieve a desired outcome including ‘forced sexual intercourse’ which is ‘unprotected’ [31, 39], and a partner who is ‘overtly overbearing or dismissive’ [31](p332).

The definitions that focus on the interpersonal level use words such as ‘control’ [18, 39, 48], ‘influence’ [33, 40], ‘power’ [40, 45, 46], ‘violence’ [18, 47, 48, 53], ‘threats’, ‘coercion’, ‘emotional blackmail’ [53], ‘force’, ‘pressure’, and ‘persuasion’ [53]. The focus of these definitions is ‘pregnancy promotion’ [44], ‘contraceptive sabotage’ [42] (including removing a condom, damaging a condom, removing a contraceptive patch, or throwing away oral contraceptives) and pregnancy outcome coercion (including influencing decisions regarding continuing with or terminating a pregnancy). A woman’s reproductive capacity was described as a ‘weapon’ that can be used against her to control reproductive outcomes [31].

These definitions suggest the goal of reproductive coercion is to ‘purposefully’ [39] ‘interfere’ with [31, 35, 49, 50], and ‘intentionally’ [38, 42] and ‘deliberately’ [18, 44] ‘dictate’ [18, 47] and ‘control’ a woman’s ‘decision-making’ and ‘autonomy’ and reduce her reproductive options including access to reproductive health services. These actions are commonly considered to be perpetrated by an individual, usually a male intimate partner or family. Some argue [18, 38, 42] reproductive coercion should be labelled as ‘reproductive coercion and abuse’ to capture the intimate partner violence dimension.

### Systems and structures

The theme *Systems and structures* describes organisational, institutional, structural and systems level forms of reproductive coercion and relates to *Freedom from external forces to achieve reproductive autonomy*. Ten of the articles in this review [33, 34, 36, 41, 43, 46, 47, 49, 52, 53] identified and described system and structure level forms of reproductive coercion. These definitions of reproductive coercion move away from an individual as a perpetrator (*Individual external exertion of control over a woman’s reproductive autonomy*) to acknowledge that reproductive coercion can also be perpetrated by ‘organised criminal activity’ [53], faith-based organisations [46], healthcare organisations [33, 41], and the state [41, 46, 49, 52]. This theme draws attention to ‘contributing factors’ including policies and lack of access to services which limit women’s reproductive autonomy [47]. These institutional systems and structures, through their attitudes, beliefs, behaviours, ‘policy and legislative contexts’ [46], are designed to establish and maintain ‘power’ and ‘control’ over a woman’s reproductive autonomy [46]. In doing so there is a ‘loss of conditions that enable autonomy’ [46](p5). Conversely, *Systems and structures* can also be ‘protective factors’ which contribute to obtaining reproductive autonomy free from coercion [47].

These understandings of reproductive coercion highlight that it is ‘bidirectional’, a ‘spectrum’ or continuum from ‘subtle to overt’, not a dichotomy, and that reproductive coercion is a ‘structural phenomenon rather than simply an interpersonal one’ [36](p8). ‘Policies that interfere with an individual’s reproductive autonomy are systems-level manifestations of coercive intimate partner violence, likely influenced by the same power dynamics and desire to exert control, and with outcomes that replicate existing socio-political inequities’ [49](p36). Often ‘threats’ to women’s reproductive autonomy are directed from those in positions of power or privilege and as such there is a ‘need for state interference against threats to women’s reproductive autonomy’ [52](p4). There is also a need to balance the level of ‘interference’ from the state and policies which can simultaneously protect and erode reproductive autonomy: ‘the lesser I am governed, the freer I am’ [52](p4).

Words such as ‘power’ to ‘establish’ and ‘maintain’ ‘control’ of women’s reproduction are used in these definitions, particularly in reference to already marginalised and othered populations (for example, women of low socioeconomic status, young women, women with disabilities, migrant women, women of culturally and linguistically diverse backgrounds, women of colour, and Indigenous women) highlighting the intersections of ‘multiple layers of structural oppression’ [52](p2). Words such as ‘freedom’ and ‘oppression’ are also used to emphasise ‘freedom as non-domination’ which systematically situates women in ‘relation to the structures that limit (or empower)’ and with ‘emphasis on the circumstances that make separate instances of oppression possible rather than on the individual and her choices’ [52](p5). This positioning highlights the need for ‘a state of being where women… are freely and equally able to participate in influencing the knowledge structures that would help shape social and institutional arrangements which would eventually influence their agency’ [52](p5).

These explanations of reproductive coercion draw attention to the inequities experienced by marginalised and othered populations which are exacerbated by ‘anti-choice reproductive health policies’, and can further reduce access to reproductive health services [49](p36). For example, jurisdictional laws can have the effect of ‘neither compel[ling] nor deny[ing] access to safe abortion’ [43](p132). Policy which, for example, legalise termination of pregnancy and enables access to services can be protective of and promote reproductive autonomy [47]. Conversely, policy as ‘the first line of defence against structural reproductive coercion must be stopping laws and regulations that force these clinics to shut down’ [49](p44) limiting women’s access to services and their reproductive options. Similarly, policy which makes reproductive health services more expensive further reduces accessibility [49] and has a greater impact on already marginalised women contributing to health disparities. However, policy that makes reproductive healthcare affordable would aid accessibility and consequently increase autonomy.

Within healthcare institutions which are governed by not only their own policies but also the policies, laws, and legislation of the state, reproductive coercion is reproduced and enacted due to their coercive effect. This is also apparent in healthcare provider actions which reflect the coercive policies of the institution and state. For example, ‘downward coercion’ whereby ‘accessible and affordable contraception’ required for reproductive autonomy is not available and simultaneously ‘upward coercion’ [36](p3) when healthcare providers do not respect women’s decisions or enable her to act on it. Two studies [34, 36] found reproductive control occurs through healthcare institutions by financially ‘incentivizing’ various methods of contraception, and not disclosing the full range of options available or providing adequate or correct information. Incentivising some types of contraception can create ‘structural incentives for providers to maximize the provision of modern methods, without any attempt to address the ways that this might incentivize coercion too’ [36](p8). Furthermore, reproductive coercion is not a singular event, rather it can occur at multiple timepoints of health care interactions [36].

These descriptions of reproductive coercion suggest the goal is to give more attention to the ‘structural’ in addition to the ‘individual’ form of coercion, recognising that reproductive coercion is a continuum. Further, to better understand the ‘everyday ways that women’ [36](p3) see their reproductive autonomy limited and to draw attention to the ways in which methods of control and coercion have been enacted, with particular focus on marginalised and othered populations of women at the hands of the state and institutions.

### Social and cultural determinants

Nine articles [32, 33, 41–43, 47−49, 52] contributed to the theme, *Social and cultural determinants* which highlights the social and cultural conditions and contexts, including concepts of gender and cultural norms, and discrimination primarily driven by dominant ideologies and discourses including racism and patriarchy. Here it becomes apparent that denial of reproductive autonomy through coercive practices is shaped by ‘misogyny’, ‘white supremacy’ and ‘neoliberalism’ [52] and structural factors which contribute to health inequity and disparity. Similar to *Systems and structures*, this theme necessitates the need for an intersectional lens in definitions of reproductive coercion. This theme demonstrates how reproductive coercion which manifests at the interpersonal level (*Individual external exertion of control over a woman’s reproductive autonomy*) is shaped by the ideologies underpinning and constituting reproductive coercion, and the social and cultural norms which act as ‘contributing factors’ to limit women’s autonomy [47].

Social and cultural understandings and practices ‘interfere with some of the most fundamental aspects of a woman’s reproductive autonomy and bodily integrity’ [52](p3) and influence reproductive decision-making. ‘Social pressures’ and ‘conservative attitudes’ coerce women into forming and acting on reproductive decisions in ways which meet or align with societal expectations, noting these expectations differ across contexts and within populations. As an example, conforming to pressure or expectations of friend’s attitudes and beliefs [48], or conservative attitudes regarding termination of pregnancy even in jurisdictions where termination is legal [49].

Social and cultural norms and values regarding women’s role in society, ‘pregnancy, motherhood, and reproduction’ [48](p1403) normalise the notion of traditional gender roles that all women can, will and want to become mothers. These ‘strictly defined gender roles place direct pressure on women’s ‘biological imperative’ to reproduce and enabled the perpetration of reproductive coercion’ [42](p15). As such, these social and cultural rules reinforce and recreate reproductive coercive practices [41] rather than disrupting the status quo. Cultural norms, for example ‘machismo’ [32, 39], which is established through gender rules and norms and enacted by men at the interpersonal level, can lead to reproductive coercion such as ‘pregnancy coercive, contraceptive sabotaging, or violent behaviors’ [32](p252).

The conditions in which a woman lives and experiences her everyday world provides the context in which reproductive decisions are made. While not necessarily direct coercive practices, these underlying conditions create indirect coercive control of a woman’s reproductive autonomy. For example, lack of access due to either resources (financial, capital, and social) or structures, transport and healthcare institutions / providers, education, housing, social exclusion, or residency or citizenship status which may afford full or restricted rights and access to women [32, 33], shape how much control a woman has over her reproductive autonomy.

### Freedom from external forces to achieve reproductive autonomy

The final theme, *Freedom from external forces to achieve reproductive autonomy*, captured the broader reproductive rights, justice, and autonomy lens identified in four of the included articles [33, 40, 43, 52], moving away from notions of ‘choice’ to consider ‘women’s status’, ‘oppression’, and ‘bodily integrity’ as factors which influence women’s reproductive decision-making. Through these lenses reproductive coercion is positioned within the broader conditions and contexts and as an artifact of these, shifting the positioning of reproductive coercion from the interpersonal violence against women context to extend the frame to the societal level. This positioning deems reproductive coercion as a ‘violation of women’s sexual and reproductive right’ [40](p157) and argues ‘systemic or structural barriers that prevent the equal participation of individuals’ [43](p130) be removed.

These explanations of reproductive coercion use words such as ‘freedoms’, ‘human rights’, ‘rights-based approach’, ‘gender-sensitive’, and ‘reproductive autonomy’, shifting away from the negative language of the definitions described in the theme *Individual external exertion of control over a woman’s reproductive autonomy*, to more positive and empowering language. Further, these definitions tend to be underpinned by human rights, which include reproductive rights, for example, the right to free choice to have a child or not, and the right to have a child ‘in a safe and healthy environment which is free from individual and state violence’ [52](p2), and ‘free from discrimination, coercion, or undue governmental influence’ [33](p12). Therefore, these definitions take a more empowering approach placing at the centre people, their rights and freedom to choose, rather than positioning individuals as the victim-survivor.

These descriptions of reproductive coercion suggest the goal is to be inclusive of the intersectionalities of women’s lives and their position in society to demonstrate that choice is constrained, and that there is ‘a duty to respect and protect individual choice in matters relating to reproduction’ which ‘requires the state to take steps to render the choice meaningful for realizing reproductive health’ for example, through the ‘provision of means to exercise choice’ [43](p129).

## Discussion

The findings of this scoping review reveal most definitions of reproductive coercion are situated within the interpersonal level, ignoring the interconnected structural, social, and cultural factors that create and perpetuate women’s reproductive coercion. This has led to a narrow definition of reproductive coercion, which does not take account of the individual within their broader setting. Defining reproductive coercion at the interpersonal level has been an important starting point in getting this public health issue recognised and on the broader health priority agenda. However, we now need to move beyond this conceptualisation to take account of the inter-related multi-level factors. Some articles included in this scoping review contend that the broader conditions beyond the interpersonal level contribute to reproductive coercion but do not constitute reproductive coercion, and thus should not be considered as part of the definition of the concept [18, 47]. However, failure to do so results in a lack of acknowledgement of the complexities of what underpins reproductive coercion and how it is produced, thus limiting the potential of interventions to address reproductive coercion. We argue that it is the structural, systems and societal level coercive practices which are then played out at the interpersonal level in the manifestation of behaviours that exert power and control over and limit women’s reproductive autonomy. The way reproductive coercion is defined has important ramifications for public health action. Furthermore, by not expanding the definition over time, the ongoing efforts for gender equality are constrained.

The analysis revealed the themes operate in an inter-related way rather than being independent, emphasising and reifying the influence of the structural and systemic contexts of reproductive coercion. For instance, dominant societal ideologies and discourses discussed in theme *Social and cultural determinants* shape the constitution and practices of organisations, institutions and systems discussed in theme *Systems and structures.* These institutions and systems in-turn create the operational conditions including policies and services in which reproductive rights, justice and autonomy are enabled and enacted (or not). Through explanations of reproductive coercion, using a reproductive justice lens, ‘intersectionality and the language of human rights to address the power asymmetries that arise due to citizenship, gender, race, caste, ethnicity, class, abilities’ [52](p4) are highlighted. As such, efforts to address reproductive coercion as defined solely at the interpersonal level will have limited impact given the broader context and conditions which intersect.

Missing from the research identified in this scoping review is reproductive coercion by commercial entities or interests. While this was implicitly discussed in *Systems and structures* in regards to ‘incentivizing’, commercial reproductive coercion was not explicit in any of the identified research included in this review. Commercial reproductive coercion may be perpetrated by healthcare providers whereby women feel coerced into agreeing to additional or more expensive treatments through fear or pressure. For example, women/couples seeking in vitro fertilisation (IVF) to conceive may feel pressured to sign up for additional treatments in fear of not conceiving if they do not. These IVF add-ons are procedures or medicines claiming to increase the chance of a pregnancy, mostly in countries where IVF is delivered in a commercial setting, including the USA and Australia [54]. While well-advertised on IVF clinics’ websites and extensively discussed on social media, these procedures often lack high-quality evidence of effectiveness and safety, and may result in unnecessary financial burdens as well as unrealistic and unfulfilled expectations [55].

### Strengths and Limitations

As with all scoping reviews, this review had its limitations. While scoping reviews do not formally appraise the quality of the evidence in the studies reviewed [26], the articles included in this scoping review did present some methodological limitations. For example, only three of the studies outlined the eligibility criteria for their sample. Further, there was limited inclusion of diverse and/or marginalised groups of women in the samples, such as migrant women and those with disability, limiting the insights and generalisability of their findings. There were also no articles that included the experiences of trans and gender diverse people who also experience reproductive coercion which meant definitions centred around cis-gender women. Therefore, future research should move beyond cis-normative and heteronormative understandings of reproductive health and coercion that privilege cis-gender, heterosexual females [56] by taking a gender transformative approach to all forms of reproductive oppression [57]. It must also be noted that the majority of the articles included came from the USA (9) and Australia (10) and therefore the context, for example policy and health system, of these countries needs to be considered when interpreting the findings.

Though a comprehensive and systematic search strategy was designed and undertaken by the research team, studies of relevance to the aim of this review may have been missed. As a result, the findings are a reflection of the analysis of the articles included in this review. However, the strengths of this review were the systematic approach taken and the use of multiple reviewers in the screening of title and abstracts and full-texts to ensure strict application of the eligibility criteria. Further, the review reported on a range of study types. Finally, this scoping review has provided important insights into the current state of how reproductive coercion is defined.

## Conclusion

The aim of this scoping review was to explore how reproductive coercion is defined, how it is defined at the social-cultural-systems-structural levels, and if the definitions of reproductive coercion are inclusive of the conditions and contexts in which reproductive coercion occurs. The identification of four interacting themes on current definitions of reproductive coercion suggests four key findings: (1) reproductive coercion is experienced in multiple ways within and across a continuum from the interpersonal to the systems, structural and societal; (2) is exercised at multiple interacting levels in an effort to diminish reproductive decision-making freedoms and choices by controlling or limiting access to reproductive options through policy, law, legislation, behaviours, attitudes and so forth; (3) is socially and culturally embedded systematic control and oppression of females reproductive rights enacted across multiple levels to hold power over female’s reproductive autonomy creating, maintaining, and reinforcing gender inequality and other intersectional forms of oppression (particularly ability, ethnicity and sexuality); and (4) manifests at the interpersonal level as an artifact of intersecting social, cultural, institutional systems and structures, and organisations and the state which set the necessary context within which reproductive coercion occurs.

We argue for a more inclusive definition of reproductive coercion that recognises the gendered nature and female’s multiple oppression and inequality. Further, reproductive coercion is, and can be, perpetrated and/or facilitated by the state and that it is produced and enacted at the societal, institutional, systems, and structural levels. We propose the following definition of reproductive coercion to capture the multiple interacting layers within which reproductive coercion occurs:

*Reproductive coercion is the act of removing or limiting reproductive autonomy to control reproductive decision-making freedoms and choices to hold power over reproductive autonomy. It occurs through socially and culturally embedded systematic control and oppression of reproductive rights, beliefs, conceptualisations of gender roles, behaviours, attitudes, and actions, practices, policy, law, and legislation resulting in gender inequality and other intersecting forms of oppression (particularly ability, ethnicity and sexuality). This manifests and is experienced at the interpersonal level in multiple ways as an artifact of interconnected and interacting forces across the social, cultural, institutional systems and structures, organisations, and the state which create the context within which reproductive coercion occurs.*

This definition is explicitly inclusive of the broader conditions and context in which reproductive coercion manifests which is required to effectively implement settings based, whole-of-population, primary prevention programs using an intersectional approach to reduce reproductive coercion and the subsequent negative health outcomes. Further research is required to explore how the ways in which reproductive coercion is defined impacts public health primary prevention approaches and outcomes, including if adopting a broader more inclusive definition of reproductive coercion would facilitate improved reproductive autonomy. This scoping review and the resulting definition have provided the important first step in situating reproductive coercion beyond the interpersonal level to allow for the primary prevention of this important public health issue.

## Electronic supplementary material

Below is the link to the electronic supplementary material.

Supplementary Material 1

## Data Availability

The datasets used and/or analysed during the current study are available from the corresponding author on reasonable request.
