# Supplementary material for: Re-defining reproductive coercion using a socio-ecological lens: a scoping review
Source: BMC Public Health. 2023 Jul 17;23:1371. doi: 10.1186/s12889-023-16281-8 (PMC10353243; doi:10.1186/s12889-023-16281-8)
Supplement: Supplementary file 1 — Supplementary Material 1 [file 12889_2023_16281_MOESM1_ESM.docx]

Supplementary Table 1: Abbreviated Data Extract Chart

| **Author(s)** | **Year** | **Country** | **Aim(s)** | **Study design** | **Sampling, recruitment, and sample size** | **Data collection** |
| --- | --- | --- | --- | --- | --- | --- |
| Bhakuni, H. | 2021 | Netherlands | To clarify theoretical understandings of reproductive justice and freedom. | Narrative review | No sampling or recruitment/no eligibility criteria provided | No data collection methods described |
| Buchanan, F., & Humphreys, C. | 2021 | Australia | To explore women's experiences of coercive control and reproductive coercion. | Qualitative | Purposive and convenience   16 women recruited through advertisements in local free press | Semi-structured interviews and focus groups |
| Dejoy, G. | 2019 | United States | To discuss historical and contemporary policies as structural forms of reproductive coercion. | Editorial | Not applicable | Not applicable |
| Douglas, H., & Kerr, K. | 2018 | Australia | To map the behaviours and concepts associated with reproductive coercion based on civil protection orders an family law responses. | Qualitative | Purposive and convenience  65 women recruited through involvement with domestic violence support workers or lawyers | Semi-structured interviews |
| Fay, K., & Yee, L. | 2018 | United States | To describe the concept of reproductive coercion. | Narrative review | No sampling or recruitment/no eligibility criteria provided | No data collection methods described |
| Grace, K.T., & Anderson, J.C. | 2018 | United States | To review the current state of knowledge regarding reproductive coercion including specific behaviours of reproductive coercion. | Systematic review | Papers included if published between 2005-2015, if focused on male partner reproductive coercion or specific behaviours of reproductive coercion, or examined sexual coercion, intimate partner violence, coercion by the government (e.g., forced sterilisation). | Searched databases: PubMed, CINAHL, PsycINFO, Embase |
| Grace, K.T., Alexander, K.A., Jeffers, N.K., Miller, E., Decker, M.R., Campbell, J., & Glass, N. | 2020 | United States | To describe the context of reproductive coercion among Latina women receiving services at an urban clinic. | Qualitative | Purposive  13 Latina women recruited from Washington health centre | Semi-structured interviews |
| Loder, C.M., Minadeo, L., Jimenez, L., Luna, Z., Ross, L., Rosenbloom, N., Stalberg, C.M., & Harris, L. | 2020 | United States | To identify the appropriate curriculum for health professionals to understand the reproductive justice movement. | Qualitative | Purposive  65 advocates and leaders of reproductive justice recruited through advocacy organisations | Delphi method |
| Mckenzie, H.A., Varcoe, C., Nason, D., McKenna, B., Lawford, K., Kelm, M.E., Opikokew Wajuntah, C., Gervais, L., Hoskins, J., Smith, K., Arkles, J., Acoose, S., & Arisman, K. | 2022 | Canada | To explore Indigenous women's experiences of, and resistance to, reproductive coercion in healthcare and social service settings. | Qualitative | Relational sampling  32 women and Two-Spirit people recruited through community forums and organisation representatives | Unstructured interviews |
| Meier, S., Sundstrom, B., DeMaria, A.L., & Delay, C. | 2019 | United States | To explore the impact of coercion on women's understanding of, and decision-making, regarding Long-Acting Reversible Contraception methods. | Qualitative | Voluntary response  79 women recruited through Facebook advertisements, online local news advertisement and flyers in local health centres | In-depth interviews and focus groups |
| Moulton, J.E., Corona, M.I.V., Vaughan, C., & Bohren, M.A. | 2021 | Australia | To explore women's experiences of reproductive coercion and abuse.  To broaden understanding of the ways reproductive coercion and abuse is perpetrated and experienced across socio-cultural contexts. | Qualitative evidence synthesis | Papers included if focused on reproductive coercion and abuse perpetrated by an intimate partner, family member or in-law, or if focused on experiences and perceptions of reproductive coercion and abuse among women. All countries included. | Searched databases: Medline, CINAHL, Embase |
| Ngwena, C.G. | 2017 | South Africa | To discuss the Convention on the Rights of Persons with Disabilities and its contribution towards affirming the reproductive autonomy of women and girls with disabilities. | Narrative review | No sampling or recruitment/no eligibility criteria provided | No data collection methods described |
| Price, L. | 2018 | Australia | To promote further understandings of, and sector responses to, reproductive coercion. | Policy submission | Not applicable | Not applicable |
| Price, E., Sharman, L.S., Douglas, H.A., Sheeran, N., & Dingle, G.A. | 2022 | Australia | To identify the proportion of women experiencing reproductive coercion and understand how reproductive coercion occurs alongside other forms of domestic violence. | Quantitative | Convenience  3,117 organisational records of women who were clients of a telephone counselling and information service for unplanned pregnancy between January 2015 and July 2017 | Client organisational data |
| Reid, S.O., Hendron, M., Currie, T., & Lee-Ack, E. | 2018 | Australia | To emphasise the impact of reproductive coercion on women's sexual and reproductive health choices, decisions and autonomy. | Policy submission | Not applicable | Not applicable |
| Rowlands, S., & Walker, S. | 2019 | United Kingdom | To describe the range of behaviours related to reproductive control. | Narrative review | Papers included if focused on heterosexual relationships, reproductive control of women by men, reproductive control as criminal activity. | Searched databases: Medline, CINAHL, PsycINFO, SocINDEX, Academic Search and British Library |
| Senderowicz, L. | 2019 | United States | To explore broader understandings of contraceptive coercion and the ways it is experienced by women. | Qualitative | Purposive  49 women recruited through key informants | In-depth interviews |
| Swan, L.E.T., Hales, T., Ely, G.E., Auerbach, S.L., Agbemenu, K. | 2021 | Sub-Saharan Africa | To investigate the psychometric properties of the Reproductive Coercion Scale among Appalachian women. | Quantitative | Voluntary response  628 Appalachian women recruited through targeted Facebook advertisements | Online survey |
| Tarzia, L. | 2018 | Australia | To discuss ways to improve health system responses to reproductive coercion. | Discussion paper | Not applicable | Not applicable |
| Tarzia, L., Wellington, M., Marino, J., & Hegarty, K. | 2019 | Australia | To explore understandings and perceptions of reproductive coercion among health practitioners. | Qualitative | Purposive  17 health practitioners recruited from an Australian public hospital via expressions of interest distributed by higher-level managers of women's health branches | Semi-structured interviews |
| Tarzia, L., Srinivasan, S., Marino, J., Hegarty, K. | 2020 | Australia | To explore and differentiate between women's experiences of "stealthing" (non-consensual condom removal) and reproductive coercion and abuse. | Qualitative | Purposive and convenience  14 women recruited from an Australian public hospital through flyers in women's health clinics | Unstructured interviews |
| Tarzia, L., & Hegarty, K. | 2021 | Australia | To discuss the conceptual clarity of reproductive coercion and abuse. | Commentary | Not applicable | Not applicable |
| Wield, T., & Bohm-Jordan, M. | 2019 | United States | To investigate why Latina women are more likely to experience reproductive coercion compared to non-Latina women. | Quantitative research proposal | Proposed recruitment via email | Online survey |
| Willie, T.C., Alexander, K.A., Caplon, A., Kershaw, T.S., Safon, C.B., Galvao, R.W., Kaplan, C., Caldwell, A., Calabrese, S.K. | 2021 | United States | To examine associations between birth control sabotage (as a form of reproductive coercion) and women's sexual risk levels. | Quantitative | Voluntary response  675 women recruited through an email invitation distributed to 11,238 Planned Parenthood patients | Online survey |
